# Not just crop or forest: an integrated land cover map for agricultural and natural areas

**DOI:** 10.1038/s41597-024-02979-w

**Published:** 2024-01-26

**Authors:** Melanie Kammerer, Aaron L. Iverson, Kevin Li, Sarah C. Goslee

**Affiliations:** 1 USDA-ARS Pasture Systems and Watershed Management Research Unit, University Park, PA 16802 USA; 2 Oak Ridge Institute for Science and Education, Oak Ridge, TN 37830 USA; 3 Department of Environmental Studies, St. Lawrence University, Canton, NY 13617 USA; 4 School for Environment and Sustainability, University of Michigan, Ann Arbor, MI 48109 USA

**Keywords:** Environmental sciences, Geography

## Abstract

Due to the key role surrounding landscape plays in ecological processes, a detailed characterization of land cover is critical for researchers and conservation practitioners. Unfortunately, in the United States, land cover data are split across thematic datasets that emphasize agricultural or natural vegetation, but not both. To address this gap, we merged two datasets, the LANDFIRE National Vegetation Classification (NVC) and USDA-NASS Cropland Data Layer (CDL), to produce integrated ‘Spatial Products for Agriculture and Nature’ (SPAN). Our workflow leveraged strengths of the NVC and the CDL to create detailed rasters comprising both agricultural and natural land-cover classes. We generated SPAN annually from 2012–2021 for the conterminous United States, quantified agreement and accuracy of SPAN, and published the complete computational workflow. In our validation analyses, we found that approximately 5.5% of NVC agricultural pixels conflicted with the CDL, but we resolved most conflicts, leaving only 0.6% of agricultural pixels unresolved in SPAN. These ready-to-use rasters characterizing both agricultural and natural land cover will be widely useful in environmental research and management.

## Background & Summary

Most agricultural landscapes include natural and semi-natural habitat, which supports biodiversity and ecosystem services critical for agricultural production^1,2^. Even small amounts of semi-natural habitat can provide disproportionate benefits for ecosystem services, including water quality, erosion mitigation, pest control, and pollination services^3–5^. Ecosystem services and the resulting benefits for agricultural production respond to both the amount and spatial configuration of agricultural and natural/semi-natural habitat patches. For example, pollinator communities vary based on area and proximity of semi-natural habitats^6^, mass-flowering crops^7^ and even abundance of flowering weeds in a previous year’s crop^8^. Conversely, agricultural habitats can have an inordinate impact on the ecological community and ecological functioning of surrounding natural areas. For instance, natural enemies, pathogens, or pesticides from agricultural areas may spill over into bordering natural habitat, thus impacting the insect communities of those areas^9–11^.

Though spatial information on surrounding land cover is increasingly important for researchers and conservation practitioners, in the United States, a single national dataset that includes both detailed natural and agricultural data does not exist. The Cropland Data Layer (CDL) is a widely used layer of land cover produced by the United States Department of Agriculture, National Agricultural Statistics Service^12^, but, for many applications, the CDL lacks sufficient detail about semi-natural and natural habitats. The CDL classifies non-agricultural habitats based on the National Land Cover Dataset^13^, which has very broad classes of semi-natural and natural habitat. For example, for the entire United States, the CDL classifies forested areas as one of three forest classes (coniferous forest, deciduous forest, or mixed forest). These broad classes obscure known variance within a region in forest composition^14^ and conveys a false equivalence between distant geographic regions (e.g. ‘mixed forest’ in California bears little resemblance to ‘mixed forest’ in Maine, but they are a single class in the CDL). Similarly, information on crop identity is necessary for many ecological and agricultural questions, but most land-cover datasets that focus on natural and semi-natural habitats represent agricultural land in broad crop classes (e.g. ‘row crop agriculture’), rather than specific crop types. For example, annual and perennial crops differ in frequency and intensity of tillage, which has significant implications for climate and soil health as intensive tillage adversely affects soil structure, chemical and biological processes, and increases emission of greenhouse gases^15,16^. Quantifying changes in agricultural land use and crop diversity also depends on land cover data that document specific crop types.

Addressing this data gap, the objective of our work was to create a national dataset that characterizes detailed classes of both agricultural and natural land cover. To accomplish our objective, we combined two national datasets of land cover, the USDA-NASS Cropland Data Layer (CDL) and the LANDFIRE National Vegetation Classification (NVC), leveraging the strengths of both products^12,17^. The CDL delineates 110 agricultural classes, allowing researchers to quantify, for example, how crop composition affects ecosystem services or biodiversity outcomes (e.g. mass-flowering canola provides abundant pollen and nectar for pollinators, while the same area of wheat has relatively few floral resources). Furthermore, because the CDL has been produced annually since 2008, researchers can examine temporal questions like typical crop rotations in a specific area, although there are some caveats and best practices for these uses of the CDL^18^. For instance, compared to reference data, the CDL historically undercounts cultivated area, but this bias has decreased over time. To characterize change in land cover, analysts must adjust estimates of crop area to account for varying bias in the CDL (see recommendations in Lark *et al*.^18^). According to Verburg *et al.*^19^, land cover is “the layer of soils and biomass, including natural vegetation, crops and human structures that cover the land surface.” We used this definition and considered specific crop types delineated by the CDL to be land cover, rather than land use, although we recognize varying definitions for land use/land cover in the literature^20^. The National Vegetation Classification is a raster product produced by the Landscape Fire and Resource Management Planning Tools (LANDFIRE) program which specifies 537 vegetation classes, of which 420 are semi-natural or natural vegetation^17^ and 23 are agricultural. Currently, the only available NVC product corresponds to vegetation status in 2016 (LF Remap, v2.0.0)^17^, but LANDFIRE has indicated plans to release additional, updated versions (LANDFIRE personnel, personal communication). As the name suggests, the NVC raster is a spatial representation of vegetation classes defined by a standard vegetation classification recently developed for the United States^21^. Specifically, the LANDFIRE NVC maps USNVC vegetation at the group level, the sixth of eight levels of USNVC hierarchy. USNVC defines group as:

A vegetation classification unit of intermediate rank (6th level) defined by combinations of relatively narrow sets of diagnostic plant species (including dominants and co-dominants), broadly similar composition, and diagnostic growth forms that reflect biogeographic differences in mesoclimate, geology, substrates, hydrology, and disturbance regimes^22^.

Compared with other national vegetation maps, LANDFIRE’s National Vegetation Classification dataset confers several advantages to users. The USNVC publishes detailed descriptions of vegetation groups including typical geographic range, vegetation structure, dominant species, and a summary of climate, soils, and environmental factors^21^. In addition to spatial products, LANDFIRE distributes a national reference database of vegetation plots with each field plot labelled as an NVC vegetation class^23^. For some vegetation types, this reference database enables users to analyze plant communities of vegetation types included in the NVC raster, facilitating spatial representation of species distributions, plant functional traits, and ecosystem services. Also, the hierarchical structure of USNVC defines topological relationships between vegetation classes, allowing users to easily reclassify LANDFIRE products to coarser vegetation types (e.g. combine USNVC groups to map vegetation at the macrogroup or division level). Mapping coarser classes of land cover typically increases classification accuracy^24^ and may be preferred for some applications.

To date, many environmental scientists have addressed the need for integrated layers of land-use by creating custom spatial datasets, which meet the needs of an immediate project but can lead to duplicated research effort and variability in final products. Geospatial data associated with specific research projects frequently cover small spatial extents, limiting reuse, and data are not commonly archived, with details of geospatial workflows being published with research results in discipline-specific journals, limiting findability. To address these challenges and reduce duplicative efforts in geospatial processing, for 2012–2021, we generated national land cover ‘Spatial Products for Agriculture and Nature’ (SPAN) and published the complete workflow necessary to update these data. We expect SPAN will be widely useful in environmental research, reducing research effort by providing a ready-to-use raster characterizing detailed classes of both agricultural and natural land cover.

SPAN has many diverse uses cases, including predicting biodiversity, ecosystem services, and climate adaption and mitigation strategies. For example, The Integrated Valuation of Ecosystem Services and Tradeoffs (InVEST) is a set of widely used spatial models that predict ecosystem services based on land cover data. InVEST predictions of crop pollination services depend on accurate characterization of agricultural and natural habitats available as one spatial product. Within broad classes of agricultural, forest, and wetland habitats, floral resources for pollinators can vary more than 250, 750, and 40-fold, respectively (Iverson *et al*. in prep), necessitating a land cover map that identifies specific types of crop and natural vegetation. We developed SPAN as an input for models of pollination services, but anticipate that models of carbon storage, crop disease, pest dynamics, and biocontrol, among other ecosystem services, will be improved by more detailed land cover data. We also foresee a variety of non-modelling uses for SPAN, such as topological analyses to identify proximity of specific crop and vegetation types. In this work we focused on improving representation of agricultural and natural land cover, as these are the primary strengths of the CDL and NVC datasets, respectively. But, based on the National Land Cover Dataset, the NVC raster includes four classes of urban/developed land. We retained these classes in SPAN, enabling users to explore questions like proximity or diversity of natural vegetation near urban areas.

In addition to our contributions to land cover and environmental science, this work supports a broader scientific movement towards data integration, sharing, and re-use^25^. Due to the rapidly increasing and large volume of available data, researchers are increasingly modifying and integrating existing datasets rather than creating entirely new data. From limnology^26^, human settlement mapping^27^, environmental health^28,29^, paleoanthropology^30^ to macroecology^31^, to name a few, scientists in many disciplines are engaged in similar efforts to standardize and integrate geospatial datasets.

## Methods

To create and verify SPAN, we developed a workflow to 1) merge a detailed map of natural vegetation in 2016 (LANDFIRE National Vegetation Classification, NVC) with a detailed, annual map of agriculture (USDA-NASS Cropland Data Layer, CDL), 2) resolve conflicting land covers and 3) produce error statistics. We applied this workflow to the NVC v2.0.0 (also called ‘2016 Remap’)^17^ and the CDL from 2012-2021^12^ for the conterminous United States. We integrated 2012–2021 CDL with the NVC from 2016 because, at present, 2016 is the only year with an NVC raster. Recent releases from LANDFIRE (LF 2019 & 2020) updated maps of vegetation attributes (e.g. vegetation cover and height), but did not affect the vegetation type products, including NVC. LANDFIRE also distributes an existing vegetation type(‘EVT’) product for 2001 and 2016, but we used NVC because it corresponds to a vetted schema classifying vegetation types^21^. Also, 2008 is the first year of the national CDL, so including the 2001 EVT vegetation map would add little value over only using a 2016 vegetation map. If future LANDFIRE releases include additional NVC maps, we anticipate updating natural vegetation in SPAN. The NVC and CDL have a 30 m spatial resolution and were available from USDA-NASS and LANDFIRE, respectively, in the NAD83 / Conus Albers projection (EPSG:5070). In their native format, NVC and CDL grids aligned, so we did not resample either dataset.

We reassigned agricultural pixels in the LANDFIRE National Vegetation Classification to a specific crop type that best matched their identity in the Cropland Data Layer. By altering only NVC agricultural classes (listed in Table 1), we ensured our merged vegetation map defined classes of non-agricultural vegetation that match the growth form specified in LANDFIRE vegetation height and cover layers. For example, pixels of ‘Laurentian & Acadian Hardwood Forest’ vegetation should correspond to pixels with tree metrics, rather than herbaceous or shrub, in the LANDFIRE layers for vegetation height and vegetation cover.

**Table 1:** Agricultural land cover classes in LANDFIRE National Vegetation Classification (NVC) we considered a match to the indicated classes in USDA-NASS Cropland Data Layer (CDL).

NVC Name	NVC Value(s)	Name(s) of Matching CDL Classes	Value(s) of Matching CDL Classes
Vineyard	7961, 7971, 7981, 7991	Grapes	69
Bush fruit and berries	7962, 7972, 7982, 7992	Blueberries, Cranberries	242, 250
Orchard	7960, 7970, 7980, 7990	Almonds, Apples, Apricots, Avocados, Cherries, Christmas Trees, Citrus, Nectarines, Olives, Oranges, Other Tree Crops, Peaches, Pears, Pecans, Pistachios, Plums, Pomegranates, Prunes, Walnuts	75, 68, 223, 215, 66, 70, 72, 218, 211, 212, 71, 67, 77, 217, 210, 76
Aquaculture	7979, 7989, 7999	Aquaculture, Open Water	92, 111
Row Crop - Close Grown Crop	7963, 7973, 7983, 7993	Fallow/Idle Cropland, Grass/Pasture, Other Hay/Non Alfalfa, All Other Crops*	61, 176, 37, various
Row Crop	7964, 7974, 7984, 7994		
Close Grown Crop	7965, 7975, 7985, 7995		
Fallow/Idle	7966, 7976, 7986, 7996		
Pasture and hayland	7967, 7977, 7987, 7997		
Wheat	7968, 7978, 7988, 7998		

*All Other Crops = Alfalfa, Asparagus, Avocados, Barley, Broccoli, Buckwheat, Cabbage, Camelina, Caneberries, Canola, Cantaloupes, Carrots, Cauliflower, Celery, Chick Peas, Clover/Wildflowers, Corn, Cotton, Cucumbers, Dbl Crop Barley/Corn, Dbl Crop Barley/Sorghum, Dbl Crop Barley/Soybeans, Dbl Crop Corn/Soybeans, Dbl Crop Durum Wht/Sorghum, Dbl Crop Lettuce/Barley, Dbl Crop Lettuce/Cantaloupe, Dbl Crop Lettuce/Cotton, Dbl Crop Lettuce/Durum Wht, Dbl Crop Oats/Corn, Dbl Crop Soybeans/Cotton, Dbl Crop Soybeans/Oats, Dbl Crop Triticale/Corn, Dbl Crop WinWht/Corn, Dbl Crop WinWht/Cotton, Dbl Crop WinWht/Sorghum, Dbl Crop WinWht/Soybeans, Dry Beans, Durum Wheat, Eggplants, Flaxseed, Garlic, Gourds, Greens, Herbs, Honeydew Melons, Hops, Lentils, Lettuce, Millet, Mint, Misc Vegs & Fruits, Mustard, Oats, Onions, Other Crops, Other Small Grains, Peanuts, Peas, Peppers, Pop or Orn Corn, Potatoes, Pumpkins, Radishes, Rape Seed, Rice, Rye, Safflower, Sod/Grass Seed, Sorghum, Soybeans, Speltz, Spring Wheat, Squash, Strawberries, Sugarbeets, Sugarcane, Sunflower, Sweet Corn, Sweet Potatoes, Switchgrass, Tobacco, Tomatoes, Triticale, Turnips, Vetch, Watermelons, Winter Wheat.

### Geospatial workflow

For each of the 48 states in the conterminous United States, we buffered a vector layer of US state boundaries^32^ by 90 m to accommodate edge effects of our raster processing. We clipped national CDL and NVC rasters to state boundaries and split each state raster into tiles of approximately 1000 km^2^ with a 90 m overlap between adjacent tiles. For each pair of CDL and NVC tiles, we executed the merge process, then joined output tiles together to generate state and national raster layers. We combined the NVC with CDL layers from 2012–2021 to create ten national annual rasters as our final output. We processed geospatial data by state to generate an archive of state rasters and facilitate joining land-use data with accuracy statistics from the CDL, which USDA-NASS publishes by state (see *Technical Validation* below).

**Fig. 1:**
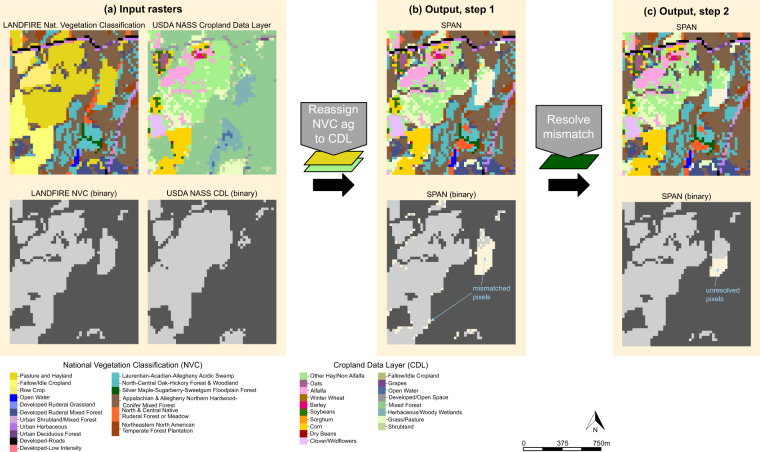
Geospatial workflow to combine USDA-NASS Cropland Data Layer (CDL) and LANDFIRE National Vegetation Classification (NVC) rasters to produce ‘Spatial Products for Agriculture and Nature’ (SPAN). Panel A shows input raster layers. Panel B shows results of reclassifying NVC agricultural pixels to CDL values. Mismatched pixels in Panel B were the result of conflicts between the NVC and CDL (see Table 1 for class matches). We resolved pixel conflicts by assigning mismatched pixels to the most common CDL agricultural class within a 3 pixel radius (Panel C). When there were no proximate CDL agricultural pixels, we could not resolve mismatch, leaving some unresolved pixels in our final SPAN rasters (Panel C). Top and bottom rows are, respectively, all classes of land cover and land cover reclassified to binary agriculture or non-agriculture. The binary representation of land cover is for visualization purposes only. The data products we produced and archived contain all classes of land cover.

We merged CDL and NVC tiles in two steps (Fig. 1). First, for pixels specified as agricultural land in NVC and CDL layers, we reassigned the NVC agricultural class to a more specific crop type from the CDL (see Table 1 for NVC/CDL class matches). We defined NVC/CDL matches based on crop classes that are synonyms (e.g. NVC wheat = CDL wheat), subsets (e.g. NVC orchard = CDL apple, peach, cherry, etc.), or potential components of a crop rotation. For example, we considered NVC row crop as a match for all annual crops (e.g. corn, soybeans, wheat, vegetables) and perennial vegetation that can be rotated with annual crops (e.g. alfalfa, hay, or pasture). In some areas of the western United States, including fallow years in crop rotations is a common strategy for moisture conservation^33^, so we considered NVC ‘Fallow-Idle Cropland’ as a match to most CDL crops (Table 1). It was necessary to consider mismatch due to crop rotations because we combined the single 2016 NVC raster with CDL data from 2012–2021, where a single CDL pixel could have multiple crop designations across the timespan.

The second step of our workflow was reassigning NVC agricultural pixels were not agriculture in the CDL (or the two agricultural class identities did not match according to Table 1). For these pixels, we identified the most likely crop type based on surrounding agricultural pixels. Specifically, we calculated the dominant CDL crop type within 90 m (7 × 7 pixel neighborhood) and assumed the focal pixel was the same crop. To account for edge effects and ensure that the dominant crop type was accurately identified from a complete 7 × 7 neighborhood, we created raster tiles that overlap by 90 m (see above). We executed the merge procedure on overlapping tiles, then clipped each tile to remove the overlapping area. If there were no agricultural pixels within 90 m of a mismatched pixel, we assigned that focal cell a value of −1001 indicating unresolved land cover due to mismatch between input rasters. In the technical validation (see below), we summarized the frequency of initial pixel mismatch between CDL & NVC input rasters (after workflow step one) and unresolved pixels remaining in merged raster (after workflow step 2).

We executed all geospatial operations in R v4.1.2^34^ using *terra* v1.5-22^35^, *raster* v3.5-15^36^, *SpaDES.tools* v0.3.10^37^, and *gdalUtils* v2.0.3.2^38^ within a Singularity container (available with code archive^39^). To facilitate processing large geographic extents, we utilized a high-performance computing cluster via the USDA-ARS SCINet Initiative. The reproducibility of this workflow ensures that future CDL years can be incorporated into SPAN updates.

## Data Records

All data files generated in this work are available through the USDA Ag Data Commons, split into two datasets for tabular and spatial files, respectively^40,41^. Contents and file formats of these datasets are as follows:

Tabular data (CSV format, available at 10.15482/USDA.ADC/1527977)^40^

1. **Attribute table for merged rasters** (CombinedRasterAttributeTable_CDLNVC.csv). This dataset is an attribute table for the merged raster product. Class names and recommended color map were taken from USDA-NASS Cropland Data Layer and LANDFIRE National Vegetation Classification. Class values are also identical to source data, except classes from the CDL are now negative values to avoid overlapping NVC values.
2. **Number and proportion of mismatched pixels** (pixel_mismatch_byyear_bycounty.csv). This dataset is the number and proportion of pixels that were mismatched between the Cropland Data Layer and National Vegetation Classification, per year from 2012–2021, per county in the conterminous United States.
3. **Number and proportion of unresolved pixels** (unresolved_conflict_byyear_bycounty.csv). This dataset is the number and proportion of unresolved pixels in the final merged rasters, per year from 2012–2021, per county in the conterminous United States. Unresolved pixels are a result of mismatched pixels that we could not resolve based on surrounding agricultural land (no agriculture with 90 m radius).
4. **Producer’s and User’s accuracy values and coverage of reference data** (accuracy_datacoverage_byyear_bycounty.csv). This dataset is values for Producer’s and User’s accuracy and coverage of reference data, per year from 2012–2021, per county in the conterminous United States. We defined coverage of reference data as the proportional area of land cover classes that were included in the reference data published by USDA-NASS and LANDFIRE for the Cropland Data Layer and National Vegetation Classification, respectively. CDL and NVC classes with reference data also had published accuracy statistics.

See Table 2 for a description of variables and column headings for each of the four tabular datasets described above.

**Table 2:** Description of variables within each of the tabular datasets we created, including specific file and column name.

Name of data resource	Name of tabular file	Column (display) name	Description
Attribute table for merged rasters	CombinedRasterAttributeTable_CDLNVC.csv	Value	Integer value of land cover class from USDA-NASS Cropland Data Layer (CDL) or LANDFIRE National Vegetation Classification. To avoid overlapping values, in merged raster, values from the CDL are opposite original values.
Attribute table for merged rasters	CombinedRasterAttributeTable_CDLNVC.csv	Class_Name	Name of land cover class from USDA-NASS Cropland Data Layer (CDL) or LANDFIRE National Vegetation Classification.
Attribute table for merged rasters	CombinedRasterAttributeTable_CDLNVC.csv	Red	Red component of color map created by USDA-NASS or LANDFIRE
Attribute table for merged rasters	CombinedRasterAttributeTable_CDLNVC.csv	Green	Green component of color map created by USDA-NASS or LANDFIRE
Attribute table for merged rasters	CombinedRasterAttributeTable_CDLNVC.csv	Blue	Blue component of color map created by USDA-NASS or LANDFIRE
Number and proportion of mismatched pixels	pixel_mismatch_byyear_bycounty.csv	FIPS	Federal Information Processing Series (FIPS) codes from U.S. Census Bureau that uniquely identify each county
Number and proportion of mismatched pixels	pixel_mismatch_byyear_bycounty.csv	State	Standard abbreviation for U.S. states
Number and proportion of mismatched pixels	pixel_mismatch_byyear_bycounty.csv	CDL_Year	Year of the USDA-NASS Cropland Data Layer (CDL)
Number and proportion of mismatched pixels	pixel_mismatch_byyear_bycounty.csv	CDL_Class	Integer value of land cover class from USDA-NASS Cropland Data Layer (CDL)
Number and proportion of mismatched pixels	pixel_mismatch_byyear_bycounty.csv	CDL_Name	Name of land cover class from USDA-NASS Cropland Data Layer (CDL)
Number and proportion of mismatched pixels	pixel_mismatch_byyear_bycounty.csv	NVC_Name	Name of land cover classes from LANDFIRE National Vegetation Classification (NVC) product with agricultural life forms. Class names were grouped to remove geographic (Western vs. Eastern) and climatic (Cool vs Warm) specifications.
Number and proportion of mismatched pixels	pixel_mismatch_byyear_bycounty.csv	NCells_Mismatch	Number of raster pixels in a given county that are mismatched between CDL and NVC
Number and proportion of mismatched pixels	pixel_mismatch_byyear_bycounty.csv	NCells_NVCClass_perCounty	Number of raster pixels in a given county for specified NVC class
Number and proportion of mismatched pixels	pixel_mismatch_byyear_bycounty.csv	NVC_CDL_Pair	Combination of ‘NVC_Name’ and ‘CDL_Name'
Number and proportion of mismatched pixels	pixel_mismatch_byyear_bycounty.csv	Pct_Mismatch	Percent of county area that is mismatched between CDL and NVC
Number and proportion of unresolved pixels	unresolved_conflict_byyear_bycounty.csv	FIPS	Federal Information Processing Series (FIPS) codes from U.S. Census Bureau that uniquely identify each county
Number and proportion of unresolved pixels	unresolved_conflict_byyear_bycounty.csv	State	Standard state abbreviation
Number and proportion of unresolved pixels	unresolved_conflict_byyear_bycounty.csv	CDL_Year	Year of the USDA-NASS Cropland Data Layer (CDL)
Number and proportion of unresolved pixels	unresolved_conflict_byyear_bycounty.csv	County	County name
Number and proportion of unresolved pixels	unresolved_conflict_byyear_bycounty.csv	LF2010_Region	Abbreviation of geographic region utilized by LANDFIRE program (see mapped regions https://www.landfire.gov/remapevt_assessment.php)
Number and proportion of unresolved pixels	unresolved_conflict_byyear_bycounty.csv	MergedRaster_Class	Value of unresolved pixels in the merged raster product
Number and proportion of unresolved pixels	unresolved_conflict_byyear_bycounty.csv	MergedRaster_ClassName	Name of unresolved pixel class in the merged raster product (also specified in raster attribute table)
Number and proportion of unresolved pixels	unresolved_conflict_byyear_bycounty.csv	NCells	Number of raster pixels in a given county that are unresolved class
Number and proportion of unresolved pixels	unresolved_conflict_byyear_bycounty.csv	Pct_Unresolved	Percent of county area that is unresolved pixels
Producer’s and User’s accuracy values and coverage of reference data	accuracy_datacoverage_byyear_bycounty.csv	FIPS	Federal Information Processing Series (FIPS) codes from U.S. Census Bureau that uniquely identify each county
Producer’s and User’s accuracy values and coverage of reference data	accuracy_datacoverage_byyear_bycounty.csv	State	Standard state abbreviation
Producer’s and User’s accuracy values and coverage of reference data	accuracy_datacoverage_byyear_bycounty.csv	CDL_Year	Year of the USDA-NASS Cropland Data Layer (CDL)
Producer’s and User’s accuracy values and coverage of reference data	accuracy_datacoverage_byyear_bycounty.csv	County	County name
Producer’s and User’s accuracy values and coverage of reference data	accuracy_datacoverage_byyear_bycounty.csv	FocalGroup	Land cover type(s) that contributed to accuracy values for the specified dataset
Producer’s and User’s accuracy values and coverage of reference data	accuracy_datacoverage_byyear_bycounty.csv	Dataset_Name	Name of the raster layer
Producer’s and User’s accuracy values and coverage of reference data	accuracy_datacoverage_byyear_bycounty.csv	NCells_County	Number of NVC pixels in a given county
Producer’s and User’s accuracy values and coverage of reference data	accuracy_datacoverage_byyear_bycounty.csv	NCells_FocalGroup	Number of NVC pixels that match focal group
Producer’s and User’s accuracy values and coverage of reference data	accuracy_datacoverage_byyear_bycounty.csv	FocalGroup_PctCounty	Percent of county area that is land cover matching the focal group
Producer’s and User’s accuracy values and coverage of reference data	accuracy_datacoverage_byyear_bycounty.csv	WithData_PctFocalGroup	Percent of focal group area that has ground truth data (e.g. CDL pixels that are land cover classes included in the CDL accuracy assessment)
Producer’s and User’s accuracy values and coverage of reference data	accuracy_datacoverage_byyear_bycounty.csv	WtdProdAcc	Mean Producer’s accuracy per county, weighted by the relative area in each land cover class
Producer’s and User’s accuracy values and coverage of reference data	accuracy_datacoverage_byyear_bycounty.csv	WtdUserAcc	Mean User’s accuracy per county, weighted by the relative area in each land cover class

Tabular datasets are available to download at 10.15482/USDA.ADC/1527977.

Spatial data (GeoTiff format, available at 10.15482/USDA.ADC/1527978)^41^

1. SPAN land cover in the conterminous United States: 2012–2021 (KammererNationalRasters.zip). This data resource includes Spatial Products for Agriculture and Nature (‘SPAN’) land cover in the conterminous United States from 2012–2021. This raster dataset is available in GeoTIFF format and was created by joining agricultural classes from the USDA-NASS Cropland Data Layer (CDL) to national vegetation from the LANDFIRE National Vegetation Classification v2.0 (‘Remap’). Pixels of national vegetation are the same in all rasters provided here and represent land cover in 2016. Agricultural pixels were taken from the CDL in the specified year, so depict agricultural land from 2012–2021.
2. Rasters of pixels mismatched between CDL and NVC: 2012–2021 (MismatchedNational.zip). This data resource is GeoTIFF rasters showing location of pixels that are mismatched between 2016 NVC and specific year of CDL (2012–2021). This dataset includes pixels that were classified as agriculture in the NVC but, in the CDL, were not agriculture (or were a conflicting agricultural class).

## Technical Validation

### Validation Methods

We validated SPAN to verify that our geospatial workflow produced the desired output and quantified spatial agreement between source layers. To ensure valid output, for all merged rasters, we verified that file size, spatial extent, coordinate reference system, and pixel values matched expected values. For expected values, see attribute table for merged raster^40^. All rasters in the linked archive^41^ passed our automated testing regime.

**Fig. 2:**
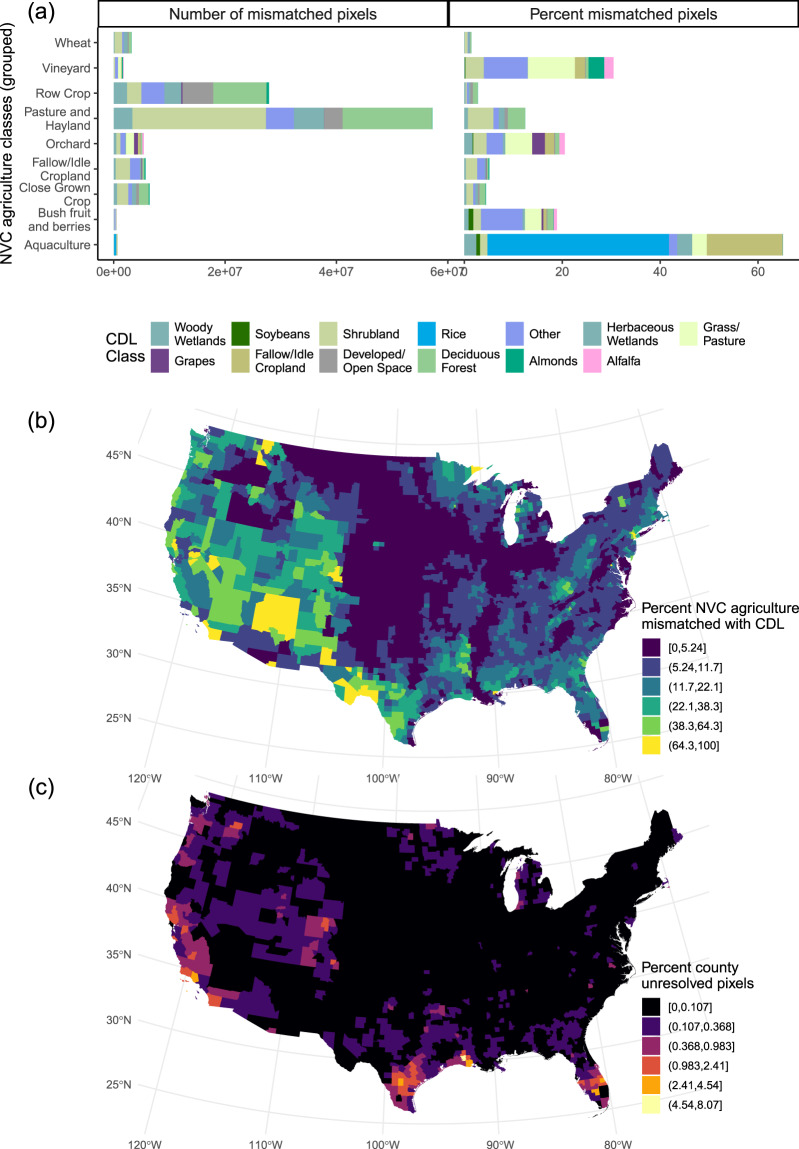
Frequency of disagreement between LANDFIRE National Vegetation Classification (NVC) agricultural classes and 2017 Cropland Data Layer (CDL). Panels A and B depict frequency of pixel disagreement in the original land-cover rasters (after step 1 of our geospatial processing, Fig. 1). Panel A shows specific CDL classes that conflicted with NVC agricultural classes and, for each county in the conterminous United States, Panel B depicts the percentage of NVC agricultural pixels that did not match CDL. For the final SPAN raster (output of workflow step 2), Panel C shows the percentage of each county where conflict between CDL and NVC layers could not be resolved. To facilitate mapping, we converted percentages to discrete intervals using Jenks natural breaks algorithm.

We quantified spatial agreement between the NVC and the 2012–2021 CDL by calculating the number and proportion of mismatched pixels present after step one of our geospatial workflow (Fig. 1). We defined mismatched pixels as NVC agricultural pixels that were not a relevant agricultural class in the CDL (Table 1). This included CDL pixels that were not agricultural (developed or natural land) or agricultural pixels that we deemed in conflict with the NVC agricultural class (see ‘Geospatial workflow’ above for rationale of class matching). For users interested in specific locations of mismatched pixels, we provided a raster dataset of all mismatched pixels in the accompanying data archive^41^. After we re-assigned values for mismatched pixels (workflow step 2), we also summarized the number and proportion of pixels with unresolved land cover in our output rasters. From preliminary analyses, we determined that there was relatively little variation in mismatched pixels over time (Figure S1, Figure S2), so we presented results for one representative year of the CDL, 2017 (Fig. 2).

We assessed error of SPAN by integrating accuracy statistics for the CDL and the NVC^42,43^. For each year and CDL class, USDA-NASS defines accuracy as the match between CDL raster and a reference dataset of administrative crop data (USDA-NASS, unpublished) and the National Land Cover Dataset^13^, another remotely sensed map of land cover. For each NVC region, LANDFIRE quantifies agreement between NVC raster and field plots in the LANDFIRE reference database^23^ labelled as specific NVC vegetation types. Due to error associated with labelling LANDFIRE field plots, ‘agreement’ rather than ‘accuracy’ is a more appropriate term for NVC assessment^42^. But, to utilize consistent language for both datasets, we referred to agreement between spatial and reference data as ‘accuracy’. To summarize accuracy of CDL, NVC, and SPAN, we calculated an area-weighted mean of accuracy data per county in the conterminous United States. We do not include a detailed description of CDL and NVC reference data because our goal was to illustrate how the classification accuracy of SPAN compares to accuracy of CDL and NVC, rather than validating the source datasets themselves. For more information on CDL and NVC validation procedures, we refer readers to CDL and LANDFIRE websites (https://www.nass.usda.gov/Research_and_Science/Cropland/sarsfaqs2.php and https://landfire.gov/remapevt_assessment.php, respectively) and Lark *et al*.^23^.

To aid interpretation of our accuracy maps, we also generated county-level maps of the proportional area of land cover classes with ground-truth data (reference data coverage) for CDL, NVC, and SPAN NVC accuracy data are only available for vegetation types with at least 30 field-surveyed plots in the LANDFIRE reference database^23^, and in some regions, many NVC classes were lacking accuracy values due to insufficient field plots. For the CDL and NVC, we calculated coverage of reference data considering only agricultural and unmanaged classes, respectively, while coverage values for SPAN included agricultural and unmanaged classes. Like our assessment of pixel mismatch, we showed data on accuracy and coverage of reference data using the 2017 CDL.

### Validation Results

In our technical validation, we found that 5.5% of NVC agricultural pixels conflicted with the 2017 CDL (i.e., the CDL was not an agricultural class or two agricultural class identities did not match according to Table 1). NVC classes for pasture/hayland and row crop had the highest number of conflicting pixels with CDL, with fewer mismatches for less common agricultural classes such as vineyard, orchard, fallow/idle, and aquaculture (Fig. 2). Like other studies^24,44^, we found classification of pasture/hayland was a challenge, with 12.4% of NVC pixels of pasture/hayland conflicting with the 2017 CDL. For other common agricultural classes in the NVC (classes totaling more than 5% of U.S. agricultural land), pixels in conflict with the CDL did not exceed 5.1% of class area (Figure S3). Agricultural classes that were less common in the NVC had fewer mismatched pixels but higher proportional mismatch with CDL. For example, 65% of aquaculture pixels in the NVC were not classified as aquaculture in the CDL, although the approximately 94,000 ha of NVC aquaculture represents only 0.05% of agricultural land in the United States. Greater than 18% of bush fruit and berries, orchard, vineyard, and aquaculture area conflicted with CDL, although total area of these classes only represents approximately 1.8% of U.S. agricultural land.

Mismatch between NVC and 2017 CDL land cover represented a median of 6.4% of county area, although mismatch was more prevalent in the Western United States. Most U.S. counties had low to moderate mismatch with less than 13% and 24% area mismatch in 77% and 90% of counties, respectively. Counties in the Western U.S. had higher mismatch, with some counties in the Southwest, California, Minnesota, Idaho, and Montana exceeding 65% of agricultural land in the NVC conflicting with the CDL. In counties with > 65% mismatch, the most common mismatches were pixels classified as pasture/hayland or orchard in the NVC and shrubland in the CDL.

By reassigning cells based on the most common agricultural land in the surrounding area, we resolved most mismatched pixels. In the final SPAN raster, most U.S. counties had very few unresolved pixels, but southern California, Texas, Louisiana along the Gulf of Mexico, and Florida were hotspots of conflicting pixels with no agricultural land within 90 m. In 96% of U.S. counties, unresolved pixels in SPAN were less than 0.4% of county area. Excepting one county in Louisiana, in hotspot areas, pixels with unresolved land cover represented 0.4–4.6% of county area. The most common cause of unresolved pixels was conflict related to classifying pasture/hayland, shrublands, wetlands, vineyard, and tree fruits. For example, in hotspots of unresolved pixels in Texas and Florida, pixels that the NVC classified as pasture/hayland were shrubland, wetland, or forest classes in the CDL. We could not resolve conflicts in these areas because there was no agricultural land within the 90 m search radius due to high proportion of non-agricultural land. In some counties in southern California, up to 4.6% of county area was unresolved land cover because 1) NVC orchard or vineyard pixels were assigned to grass/pasture or shrubland in the CDL or 2) NVC row crop or vineyard pixels were tree nuts (almonds, walnuts, or pistachios) in the CDL. When conflicting land cover in California included orchard or vineyard classes, unresolved pixels typically represented a whole field (large, regular shape). If users are focusing on counties with more unresolved pixels, we recommend collecting ground-truth data or consulting additional imagery or land cover data to resolve mismatch between CDL and NVC layers.

The LANDFIRE program uses the CDL to classify broad agricultural types in the NVC, and one aspect of the LANDFIRE workflow likely contributed to mismatched pixels between the NVC and the CDL. In previous years, CDL models sometimes generated noise pixels scattered across a field that were misclassified as other crops. To address this, LANDFIRE executed a zonal smooth reclassifying CDL to the majority crop type within polygons of ownership. Then, LANDFIRE utilized the smoothed CDL to define agricultural classes for NVC (LANDFIRE program, personal communication). We could not replicate the LANDFIRE methodology because the spatial layer of ownership polygons (Common Land Unit) is not publicly available^45^ but, when CDL and NVC conflicted, we also determined crop identity from surrounding crop pixels. Also, in recent years, USDA-NASS improved within-field classification of the CDL and, starting with a 2020 update to LANDFIRE products depicting fuels, fire, and vegetation height/cover, LANDFIRE dropped the zonal smooth from their workflow (LANDFIRE program, personal communication).

At a national scale, classification accuracy of the CDL was higher than the NVC and accuracy of SPAN was between the 2017 CDL and NVC values because we calculated SPAN accuracy as a weighted average of accuracy of the input layers. For both input layers and SPAN, we calculated accuracy per county yielding a distribution of accuracy values (Figure S4). For user’s and producer’s accuracy of the 2017 CDL, the median of county accuracy values was 75.8% and 81.5%, respectively, compared with 49.5% and 51% for the NVC (Figure S4). For SPAN, median user’s accuracy was 57% and producer’s accuracy was 56.7%. Accuracy of SPAN fell between 2017 CDL and NVC values (Figure S4), albeit closer to the NVC due to high proportion of natural land in many counties.

**Fig. 3:**
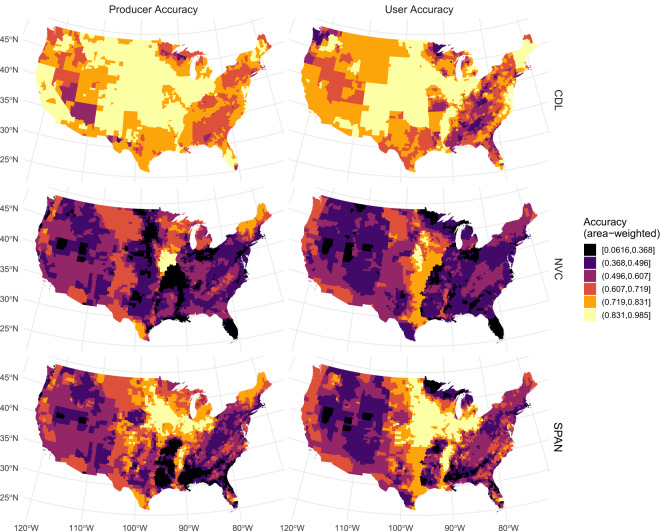
For 2017 CDL, NVC, and SPAN (NVC + CDL), accuracy of land cover classification compared against reference data. Accuracy statistics were calculated per county as an area-weighted mean of accuracy values assigned to each land cover class. For visualization purposes, we converted accuracy values to discrete intervals using Jenks natural breaks algorithm.

Accuracy of the CDL, NVC, and SPAN varied geographically. The 2017 CDL was most accurate in high agricultural areas, especially the Great Plains and much of the Midwestern Corn Belt, with lower accuracy in the Mid-Atlantic, Southeast, Upper Midwest, Pacific Northwest, and Southwest regions (Fig. 3). For the NVC, we did not find any relationship between accuracy and the amount of natural land in each county, likely because large areas of natural land can comprise many habitat types, while large swaths of agriculture are typically simplified landscapes with relatively few crop types. We found a regional hotspot of user’s accuracy exceeding 72% from Iowa and western Missouri south to central Texas (Fig. 3). User’s and producer’s accuracy of the NVC were both low (<37%) in Florida, eastern Missouri, and some areas in Ohio and Indiana. In regions including Florida, western Texas, and Missouri and Arkansas, the LANDFIRE program had very little reference data and was not able to assess accuracy of many vegetation types (see coverage of reference data below). Even for vegetation types included in the accuracy assessment, the resulting accuracy values were likely lower due to the lack of reference data from these areas. Accuracy of SPAN most closely resembled the NVC, with higher accuracy in the Midwestern Corn Belt due to prevalence of agriculture in this region (Fig. 3). It would be interesting to analyse relationships between environmental and vegetation characteristics and classification accuracy, but this was beyond the scope of the current work.

**Fig. 4:**
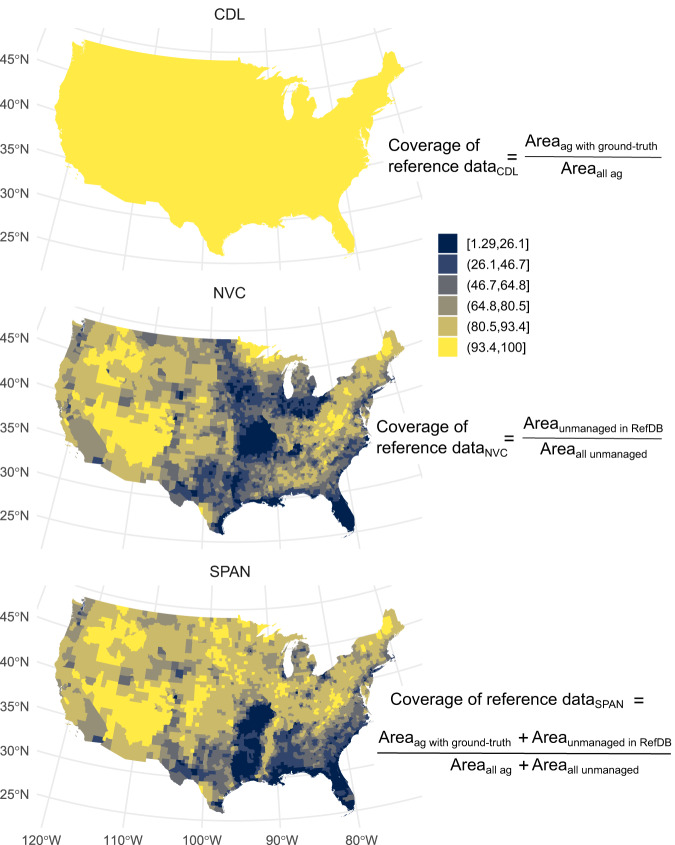
Percentage of county area with reference data for the conterminous United States. For 2017 CDL, reference data were from a USDA administrative crop database and National Land Cover Dataset imagery. For NVC, reference data were field plots in LANDFIRE reference database, with a minimum of 30 field plots per NVC class. For SPAN, we calculated coverage of reference data as a fraction of agricultural and unmanaged classes, respectively, while, for the merged product, coverage of reference data included agricultural and unmanaged classes. To facilitate mapping, we converted values to discrete intervals using Jenks natural breaks.

We found that coverage of reference data for the CDL was consistently high, but, for the NVC (and therefore SPAN), coverage ranged from 1.3% to nearly 100% by county (Fig. 4, Figure S5). For CDL, coverage of reference data was > 93% for the whole conterminous United States because the CDL accuracy assessment included nearly all crop classes. For the NVC and SPAN, median coverage was 77.2% and 84.4%, respectively. Most counties with NVC reference coverage less than approximately 50% were in the Midwest, Texas, Florida, and coastal areas along the Gulf of Mexico and Atlantic Ocean (Fig. 4). In Florida, western Texas, and Missouri and Arkansas, reference coverage for the NVC was low in many counties that had moderate to high proportional area of natural vegetation (Figure S6). This suggests that even relatively abundant vegetation types in these areas had insufficient reference data to be included in LANDFIRE’s accuracy assessment. Lack of reference data in these areas likely also contributed to low accuracy values. For the NVC, reference coverage less than approximately 30% corresponded to lower accuracy values (Figure S7). Like our accuracy results, for SPAN, spatial patterns of reference coverage resembled NVC with higher values in the Midwestern Corn Belt (Fig. 4).

## Usage Notes

We created a new spatial dataset that integrates both agricultural and natural land cover and that can be used by environmental scientists and managers who are interested landscape-scale processes involving both land cover categories. Though we were able to reduce additional inaccuracies from artifacts of our geospatial processing, the product we created is still subject to limitations of the source data. Accuracy of CDL is highest in regions dominated by agriculture and lower in states with more developed or semi-natural land cover, particularly mixed-use landscapes^24,43^. LANDFIRE vegetation products like the NVC are designed to be used at landscape, regional, or national scales and are likely less accurate for very small spatial extents^42^. For rare vegetation classes, LANDFIRE does not publish data on agreement between field plots and NVC raster. If projects are targeting small spatial extents or rare vegetation types, we recommend users review our land cover maps and adjust based on local knowledge or additional vegetation surveys.

SPAN is based on a static representation of natural vegetation circa 2016, so likely contains errors for vegetation types or geographic regions that are rapidly changing or experience frequent disturbance. By including CDL for 2012–2021, we captured the dynamic nature of agricultural land cover due to crop rotations, but the total amount of natural vs. agricultural land is fixed based on land cover in 2016. Consequently, SPAN is not appropriate for analyses of natural land conversion to agriculture or vice versa. With future releases of the NVC, we hope to more frequently update natural vegetation in SPAN to better align with other common LULC products (e.g. 5-year release schedule of the National Land Cover Database)^13^.

### Supplementary information

Supplementary Figures

## Data Availability

We archived all code to generate our merged dataset on Zenodo^39^ (available at 10.5281/zenodo.6803199) to facilitate updating these spatial layers with new versions of LANDFIRE NVC or additional years of CDL.
