# Supplementary material for: Not just crop or forest: an integrated land cover map for agricultural and natural areas
Source: Sci Data. 2024 Jan 26;11:137. doi: 10.1038/s41597-024-02979-w (PMC10817889; doi:10.1038/s41597-024-02979-w)

Figure S1: Frequency of disagreement between LANDFIRE National Vegetation Classification (NVC) agricultural classes and 2012-2021 Cropland Data Layer (CDL). These data are frequency of pixel disagreement in the original land-cover rasters (after step 1 of our geospatial processing, Figure 1). For simpler visualization, we grouped original NVC classes according to land use, collapsing temperature and geographic distinctions (e.g. western cool temperate row crop, eastern cool temperate row crop, western warm temperate row crop, eastern warm temperate row crop were classified as ‘row crop’). Here we show specific CDL classes that conflicted with NVC agricultural classes.


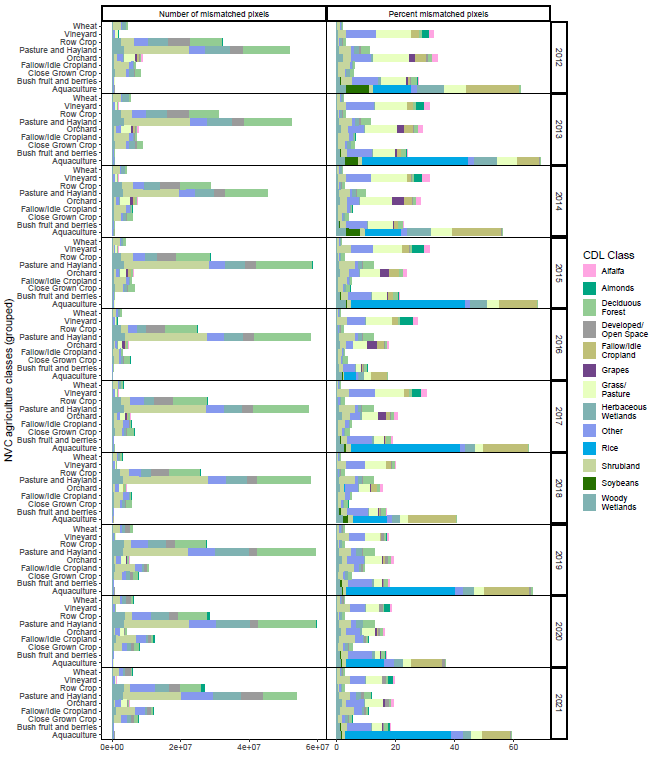


Figure S2: Spatial patterns in disagreement between LANDFIRE National Vegetation Classification (NVC) agricultural classes and 2012-2021 Cropland Data Layer (CDL). These data are frequency of pixel disagreement in the original land-cover rasters (after step 1 of our geospatial processing, Figure 1). For each county in the conterminous United States, we show percentage of NVC agricultural pixels that did not match CDL. To facilitate mapping, we converted percentage of mismatched pixels to discrete intervals using Jenks natural breaks algorithm.
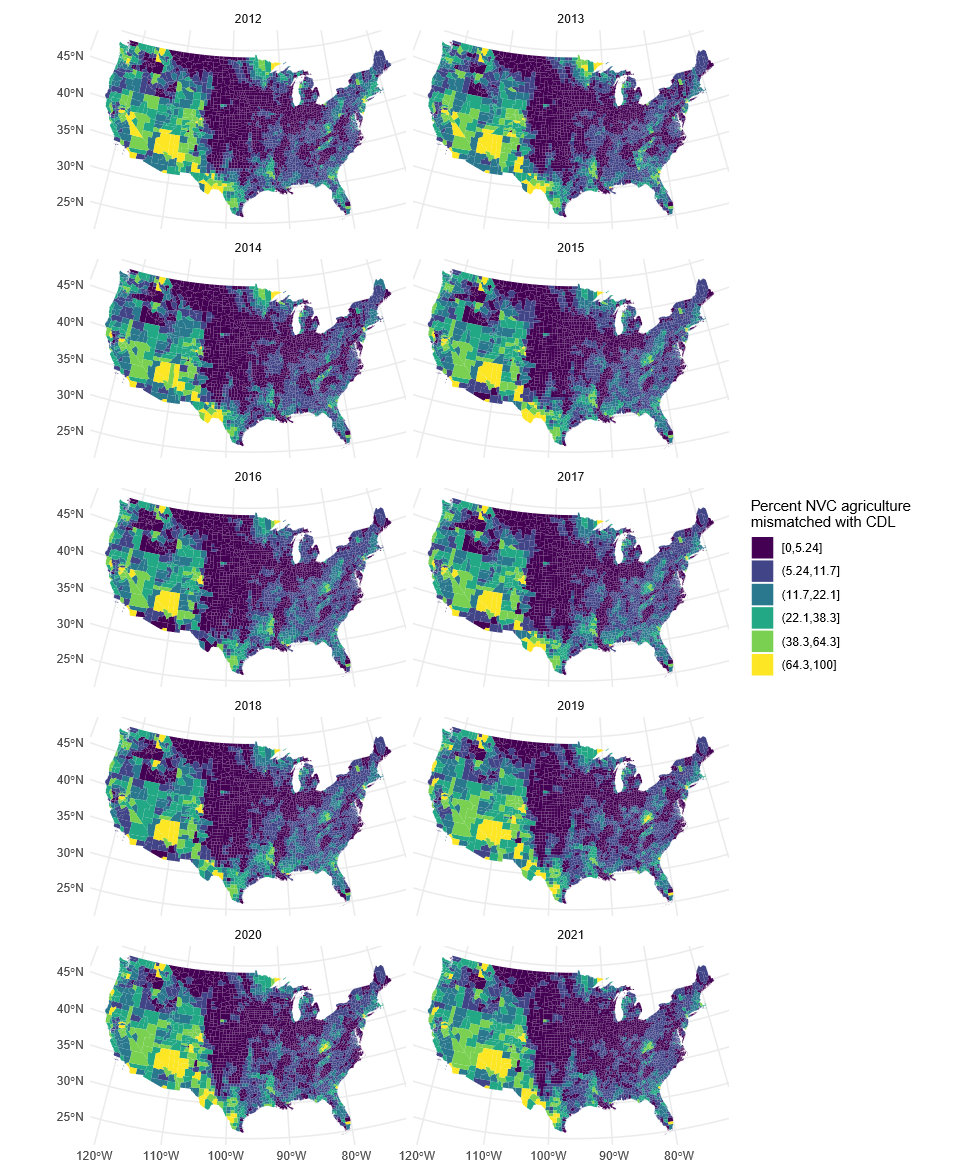


Figure S3: Area of LANDFIRE National Vegetation Classification (NVC) agricultural classes as a proportion of all agricultural land in the United States versus the proportion of NVC agricultural pixels that mismatch with CDL. For simpler visualization, we grouped original NVC classes according to land use, collapsing temperature and geographic distinctions (e.g. western cool temperate row crop, eastern cool temperate row crop, western warm temperate row crop, eastern warm temperate row crop were classified as ‘row crop’).


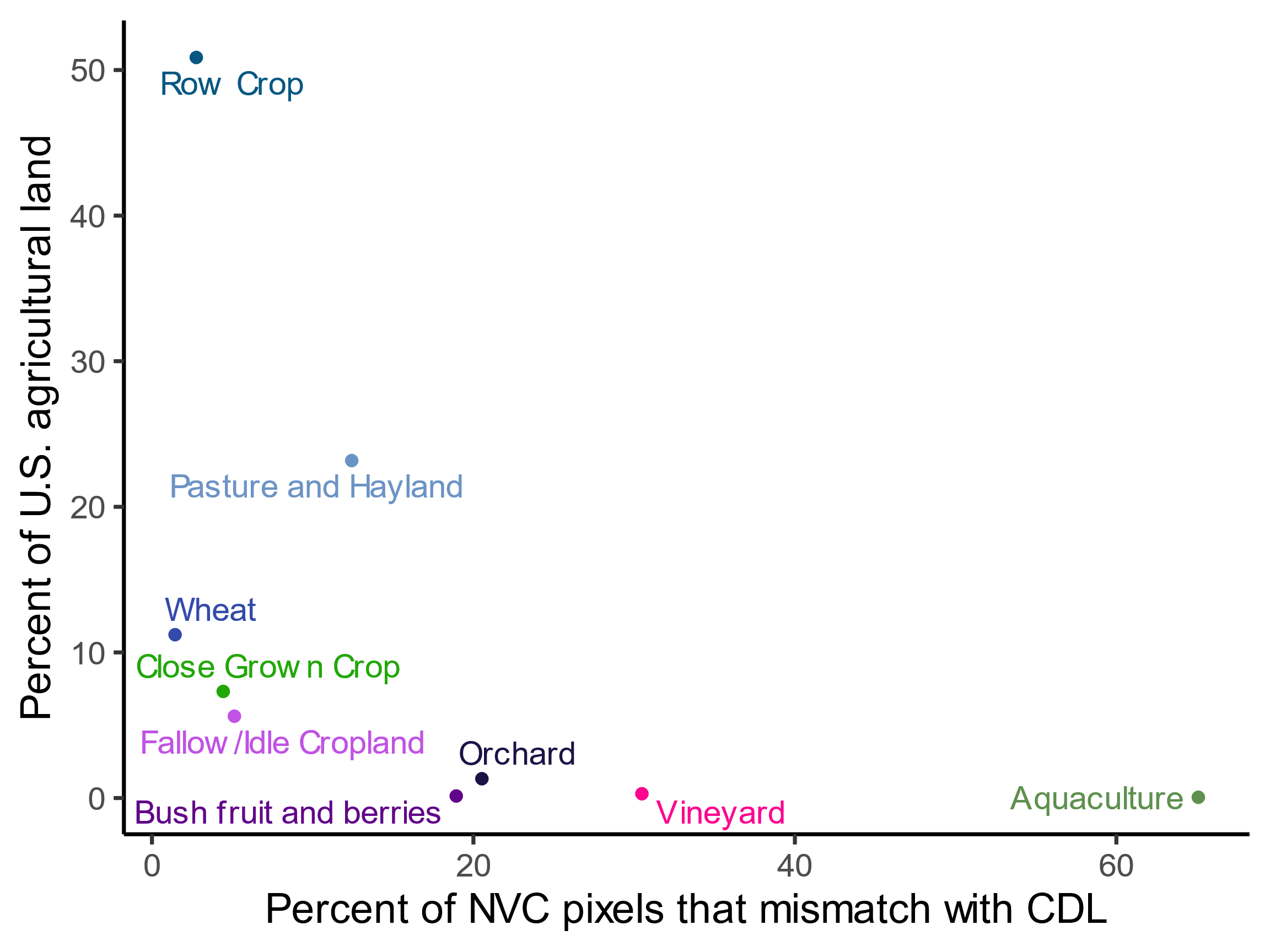


Figure S4: Distribution of producer’s and user’s accuracy for each county in the 2017 Cropland Data Layer (CDL), LANDFIRE National Vegetation Classification (NVC), and our merged raster (NVC+CDL). We calculated accuracy values per county in the conterminous United States as the mean of accuracy values for each land cover class weighed by the class area.


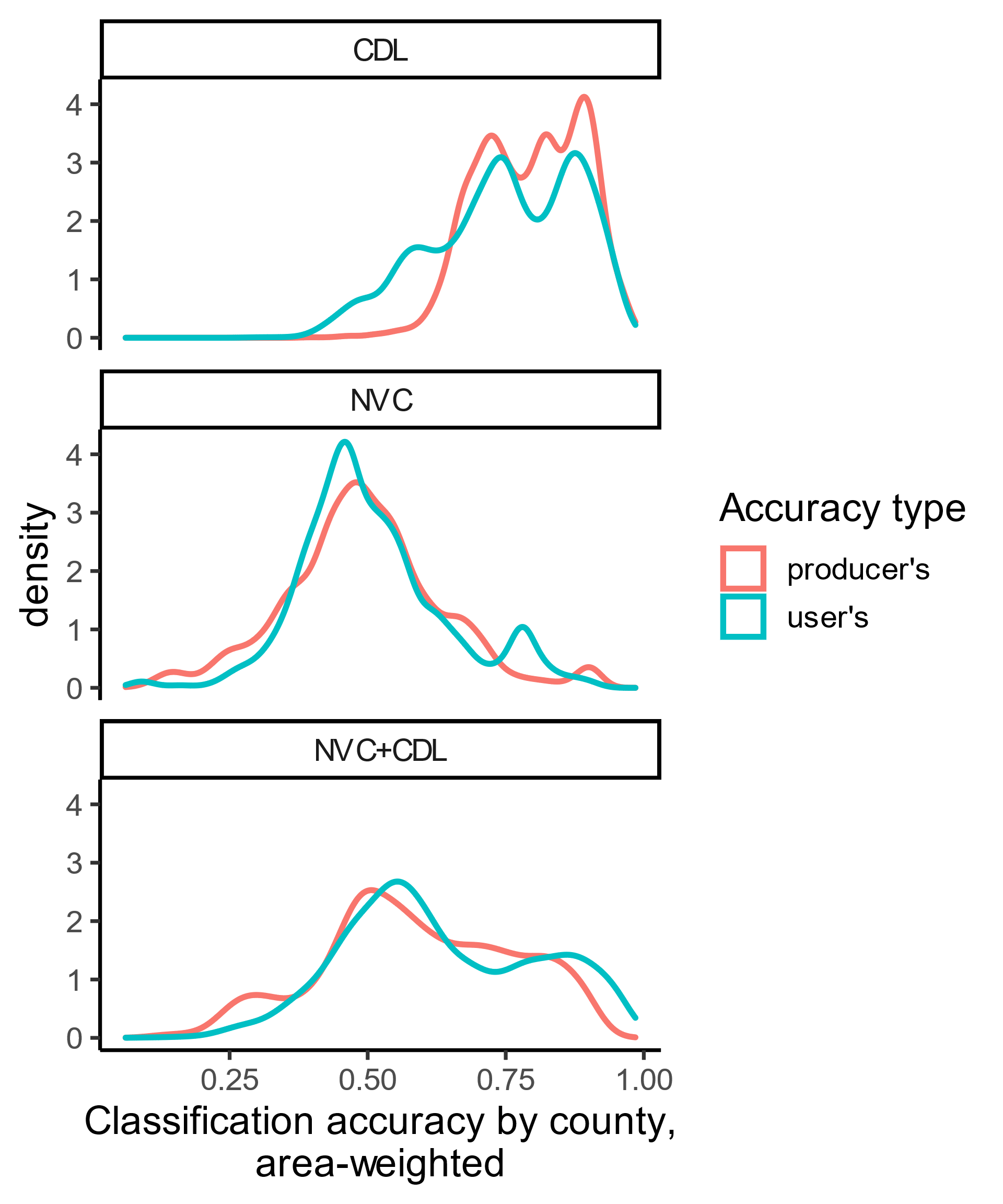


Figure S5: Distribution of coverage of reference data the 2017 Cropland Data Layer (CDL), LANDFIRE National Vegetation Classification (NVC), and our merged raster (NVC+CDL). We calculated reference coverage for each county in the conterminous United States. For CDL, reference data were from a USDA administrative crop database and imagery from the National Land Cover Dataset. For NVC, reference data were field plots in LANDFIRE reference database, with a minimum of 30 field plots per NVC class. For CDL and NVC, respectively, we calculated coverage of reference data as a fraction of agricultural and unmanaged classes, while, for the merged product, coverage of reference data included agricultural and unmanaged classes.

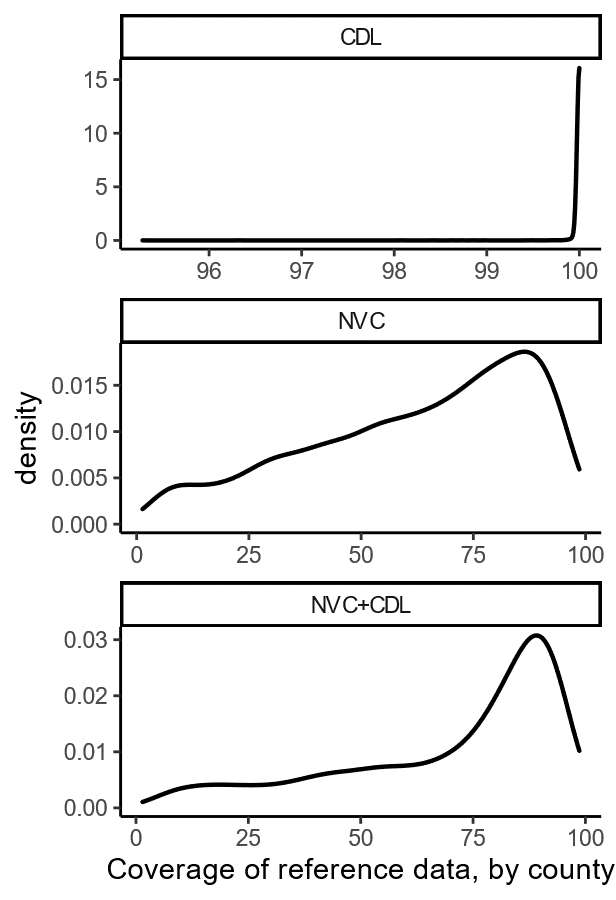


Figure S6: Spatial pattern of reference coverage and amount of agricultural and natural land for the 2017 Cropland Data Layer (CDL) and LANDFIRE National Vegetation Classification (NVC) rasters. Values were calculated per county for the conterminous United States. For visualization purposes, we condensed percentage of land cover and reference coverage to low, medium, and high classes.


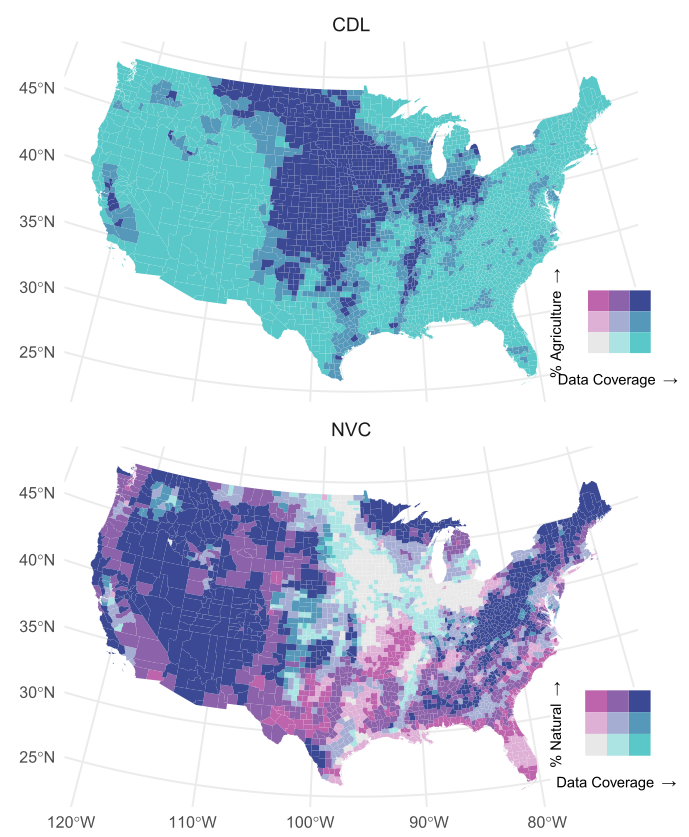


Figure S7: Relationship between coverage of reference data and accuracy for the 2017 Cropland Data Layer (CDL), LANDFIRE National Vegetation Classification (NVC), and our merged raster (NVC+CDL). We calculated all values at the county level for the conterminous United States.

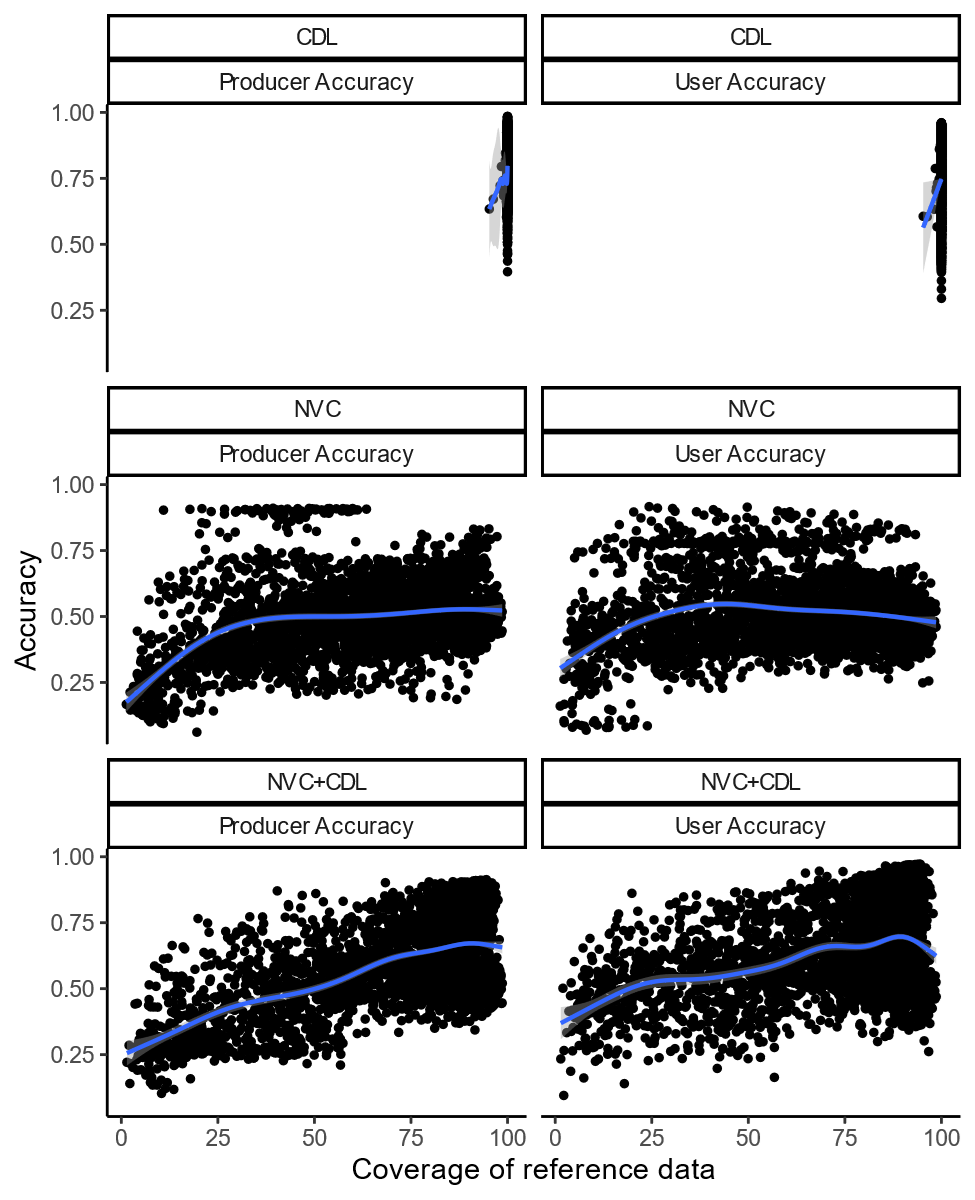

Supplement: Supplementary file 1 — Supplementary Figures [file 41597_2024_2979_MOESM1_ESM.docx]
